# Patients with Infections of The Central Nervous System Have Lowered Gut Microbiota Alpha Diversity

**DOI:** 10.3390/cimb44070200

**Published:** 2022-06-29

**Authors:** Marta Grochowska, Tomasz Laskus, Marcin Paciorek, Agnieszka Pollak, Urszula Lechowicz, Michał Makowiecki, Andrzej Horban, Marek Radkowski, Karol Perlejewski

**Affiliations:** 1 Department of Immunopathology of Infectious and Parasitic Diseases, Medical University of Warsaw, 02-106 Warsaw, Poland; marta.grochowska@wum.edu.pl (M.G.); mradkowski@wum.edu.pl (M.R.); 2 Department of Adult Infectious Diseases, Medical University of Warsaw, 01-201 Warsaw, Poland; tlaskus@yahoo.com (T.L.); mpaciorek@op.pl (M.P.); michalmakowiecki08@gmail.com (M.M.); ahorban@zakazny.pl (A.H.); 3 Department of the Medical Genetics, Medical University of Warsaw, 02-106 Warsaw, Poland; apollak@wum.edu.pl; 4 Department of Genetics and Clinical Immunology, National Institute of Tuberculosis and Lung Diseases, 01-138 Warsaw, Poland; ulka100@gmail.com

**Keywords:** neuroinfection, encephalitis, gut microbiome, 16S rRNA sequencing

## Abstract

There are multiple lines of evidence for the existence of communication between the central nervous system (CNS), gut, and intestinal microbiome. Despite extensive analysis conducted on various neurological disorders, the gut microbiome was not yet analyzed in neuroinfections. In the current study, we analyzed the gut microbiome in 47 consecutive patients hospitalized with neuroinfection (26 patients had viral encephalitis/meningitis; 8 patients had bacterial meningitis) and in 20 matched for age and gender health controls. Using the QIIME pipeline, 16S rRNA sequencing and classification into operational taxonomic units (OTUs) were performed on the earliest stool sample available. Bacterial taxa such as *Clostridium*, *Anaerostipes*, *Lachnobacterium*, *Lachnospira*, and *Roseburia* were decreased in patients with neuroinfection when compared to controls. Alpha diversity metrics showed lower within-sample diversity in patients with neuroinfections, though there were no differences in beta diversity. Furthermore, there was no significant change by short-term (1–3 days) antibiotic treatment on the gut microbiota, although alpha diversity metrics, such as Chao1 and Shannon’s index, were close to being statistically significant. The cause of differences between patients with neuroinfections and controls is unclear and could be due to inflammation accompanying the disease; however, the effect of diet modification and/or hospitalization cannot be excluded.

## 1. Introduction

Disruptions in the microbiota–gut–brain axis and gut dysbiosis are common in Parkinson’s disease (PD) [1,2], Alzheimer’s disease (AD) [3], and multiple sclerosis (MS) [4,5], and there is some evidence from clinical studies and experimental animal models that they could play some role in depression [6], autism spectrum disorder (ASD) [7], insomnia [8], and disturbance in the circadian rhythm [9]. The principal link between the gut bacterial community and its either protective or harmful effect on CNS function is ascribed to brain microglia playing both neuro- and immune functions [10,11].

Despite extensive microbiome research in various neurological disorders, the gut microbiome was so far not analyzed in infectious encephalitis/meningitis. However, a large study on autoimmune encephalitis did not find any significant differences in gut microbiome between anti-NMDA receptor (NMDAR)-positive patients and healthy controls in both alpha (within sample diversity) and beta diversity (comparison of diversity between ecosystems); [12].

Neuroinfections remain a considerable cause of mortality and morbidity worldwide [13]. However, because of a large number of potential pathogens, their typical transient presence and low copy number in the cerebrospinal fluid (CSF), as well as difficulties in pathogen culture and delayed serological response, the causative agent remains unidentified in 63–85% of patients [14,15,16,17,18].

In the current study, we analyzed the gut microbiome in 47 consecutive patients with a clinical diagnosis of neuroinfection (26 patients had viral encephalitis/meningitis and 8 patients had bacterial meningitis) and in 20 controls. The goal of the study was to determine whether patients with neuroinfection have any gut microbiota changes when compared to healthy subjects and whether the etiology (viral and bacterial) is reflected by differences in the gut microbiome.

## 2. Materials and Methods

### 2.1. Patients

The study group comprised 47 consecutive adult (≥18 years old) patients (24 women, 23 men) with a diagnosis of neuroinfection who were admitted to The Warsaw Hospital for Infectious Diseases between May and December 2019. Diagnosis of encephalitis was based on neurological manifestations, including a decreased level of consciousness and/or focal neurological signs and at least one abnormality of the CSF (white blood cell count ≥ 4 cells/mm^3^ or protein level ≥ 40 mg/dL). Meningitis was diagnosed when meningeal signs were coupled with the above CSF abnormalities; when blood cell count ≥ 300 cells/mm^3^ with a predominance of polymorphonuclear cells and/or protein level ≥ 200 mg/dL, bacterial meningitis was assumed. None of the patients had a history of any other medical condition or recent infections which could have had an effect on microbial composition.

The most commonly observed symptoms and signs are listed in Table 1. The chief complaint was headache (78.72%), followed by fever (65.96%), and focal neurological signs, which were observed in 40.43% of patients (Table 1). The average total cell count in CSF was 190 cells/mm^3^ with a dominance of lymphocytes (63%), and a mean concentration of protein was 1.51 g/L (Table 1).

Twenty-three (48.94%) patients were treated with antibiotics prior to the collection of the stool sample, but none earlier than 3 days before hospital admission. Ceftriaxone was the most commonly used antibiotic either alone (in 3 patients) or in combination with Vancomycin (2 patients); the remaining patients were treated with Vancomycin, Cloxacillin, Meropenem, Penicillin, Clarithromycin, Ampicillin, and Azithromycin. Additionally, some patients were treated with Doxycycline, Phenoxymethylpenicillin, Clindamycin, Amoxicillin, and Clavulanic acid. Antiviral treatment with Aciclovir was administered in 15 patients, and 7 patients received antiepileptic drugs.

Based on CSF analysis, viral encephalitis/meningitis was diagnosed in 26 patients, but a specific pathogen was identified in only 9 patients: 7 patients had tick-borne encephalitis virus (TBEV) and 2 patients had varicella-zoster virus (VZV) infection. The causative agent was undetermined in 17 cases (36%). Bacterial meningitis was diagnosed in eight patients, with the offending pathogen identified in 5 patients (*Neisseria meningitidis* in 2 cases, *Haemophilus influenzae*, *Streptococcus pneumoniae*, and *Staphylococcus epidermidis* in one case each). In 13 patients (27.66%), the distinction between viral and bacterial neuroinfection could not be made.

The control group consisted of twenty healthy subjects matched for age and sex. Controls did not receive any antibiotics within the last 6 months. Demographical, clinical, and laboratory data of patients and controls are shown in Table 1.

### 2.2. Stool Samples

Fecal samples (approx. 0.5 g) were collected from patients during their first defecation after hospital admission (within 24 h after admission and up to 5 days after first symptoms) into standard sterile stool collection tubes, which were immediately frozen and stored at −80 °C until processing. Control subjects collected stool at home into identical tubes, which were frozen within 2 h and stored at −80 °C.

### 2.3. Pathogen Identification

Herpes simplex virus 1 (HSV-1), herpes simplex virus 2(HSV-2), VZV, enteroviruses (EV), and human parechovirus (HPeV) were detected in CSF by the Bosphore Viral Meningitis Panel Kit (Anatolia Geneworks, Istanbul, Turkey) combined with CFX96 Real-Time System (Bio-Rad Laboratories Inc., Hercules, CA, USA). Additionally, all CSF samples were tested using in-house assays, which were previously successfully used for the identification of viral genomic DNA/RNA in CSF [16]. In short, RNA was extracted from 200 μL of CSF by Trizol LS (Thermo Fisher Scientific, Waltham, MA, USA), whereas DNA was isolated from 200 μL of CSF by NucleoSpin Plasma XS kit (Macherey Nagel, Düren, Germany) and eluted in 20 μL of water. RNA was reversely transcribed (RT) using random primers and M-MLV Reverse Transcriptase (20U). The following viruses were tested for: HSV-1/2, VZV, Epstein-Barr virus (EBV), cytomegalovirus (CMV), human herpesvirus type 6 (HHV-6), human adenoviruses (HAdVs), and EVs (Coxsackie A9, A16, B2, B3, B4, B5; ECHO 5, 6, 9, 11, 18, 30; and EV 71). Anti-TBEV IgM antibodies were detected in blood and CSF by Anti-TBE Virus ELISA (Euroimmun, Lübeck, Germany).

Patients with suspected neuroborreliosis were tested using ELISA test Borrelia IgM Rekombinant and Borrelia IgG Recombinant (Biomedica, Vienna, Austria). Positive results were confirmed by western blot test recomLine Borrelia IgM and recomLine Borrelia IgG (Mikrogen Diagnostik, Neuried, Germany). All CSF samples underwent standard bacterial cultures.

### 2.4. 16S rRNA Library Preparation and Sequencing

Bacterial DNA was extracted from 180–220 mg of stool using Nucleospin DNA Stool Kit (Macherey-Nagel, Düren, Germany) following the manufacturer’s protocol and suspended in 100 μL of elution buffer. DNA was measured using Qubit High Sensitivity Kit (ThermoFisher, Waltham, MA, USA). Next, V3 and V4 regions of the 16S rRNA gene were amplified (550 bp product) using 12 ng of bacterial DNA, V3-V4 primers [19], and 0.5U KAPA HiFi HotStart ReadyMix PCR Kit (Roche Molecular Diagnostics, Basel, Switzerland). PCR products were purified using a 0.8 ratio of AMPure XP beads (Beckman Coulter Life Sciences, Brea, CA, USA) and their quality was determined using Bioanalyzer and DNA 1000 kit (Agilent Technologies, Santa Clara, CA, USA). Sequencing libraries were dual-indexed using Nextera XT Index kit (Illumina, San Diego, CA, USA) and purified using a 1.1 ratio of AMPure XP beads (Beckman Coulter Life Sciences, Brea, CA, USA). All samples were normalized and sequenced (300 nt, paired-end reads) using MiSeq Reagent Kit v3 (600-cycle) on Illumina MiSeq platform.

### 2.5. Bioinformatics and Statistics

Quality control of raw reads was performed using FastQC software [20]. Reads were trimmed using trimmomatic [21] and, based on their size, selection was performed using BBTools [22]. Reads were further analyzed with QIIME version 1.9.1 [23]. In short, forward and reverse reads were merged using the fastq-join command [24]. Operational taxonomic units (OTUs) were determined by an open-reference OTU picking process in which reads were clustered against reference sequences (threshold value of 97% sequence similarity), and any unmapped reads were subsequently clustered de novo. Chimera detection was carried out using ChimeraSlayer [25]. Normalization was performed with the use of metagenomeSeq’s CSS (cumulative sum scaling) transformation [26].

Statistical analysis was performed on alpha and beta diversity metrics. Principal Coordinates Analysis (PCoA) plots (unweighted and weighted UniFrac data) for beta diversity were generated using QIIME data in PhyloToAST [27]. Abundance plots were prepared using the phyloseq package in R [28]. The Mann–Whitney U test was used for alpha diversity statistics, whereas taxonomical comparison between different groups was performed using a nonparametric t-test. Additionally, categorical variable analysis of similarities (ANOSIM) was performed.

## 3. Results

**Figure 1:**
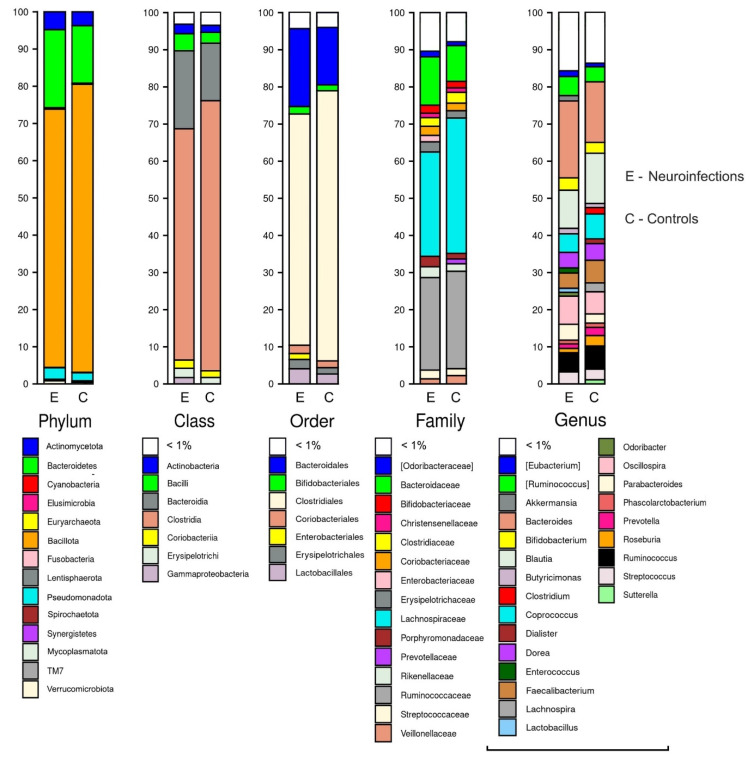
Relative abundance above 1% (all *Phylum* taxa were visualized) at different bacterial taxonomic levels in patients with neuroinfection and controls.

The number of 16S rRNA sequencing reads in patients with neuroinfections was 18,072–48,668, with a mean of 37,085 sequences per sample, whereas in controls, the number of sequences ranged from 20,863 to 53,590 (mean 40,010). Results of the open-reference OTU picking process using a normalized dataset are shown in Figure 1.

After normalization, 14 and 12 bacterial *phyla* were identified in neuroinfections and control patients, respectively. Representatives of *Bacillota* were significantly less abundant in patients than in controls (*p* = 0.015) and this was also true for both viral (*p* = 0.037) and bacterial etiology (*p* = 0.037). Moreover, bacterial meningitis had a significantly higher abundance of *Actinomycetota* (*p* = 0.037) compared to controls, whereas *Verrucomicrobiota* (*p* = 0.03) was more abundant in viral cases than in healthy subjects (Table 2). At the *Class* level, there were differences in *Clostridia*, *Coriobacteriia*, and *Verrucomicrobiae* (Table 2). A lower abundance of *Clostridiales* was observed in all patients with neuroinfection when compared to controls, whereas *Coriobacteriales* were significantly more prevalent in the latter group. At the Family level, there were no significant differences, with the only exception being increased ratios of *Eubacteriaceae* and *Verrucomicrobiaceae* in viral cases when compared to controls. Six bacterial genera were less abundant in the whole neuroinfection group and also in viral etiology cases when compared to controls. There were no differences in genera between bacterial meningitis and healthy subjects. Similarly, there were no statistically significant differences in gut microbial composition across any taxonomic level between viral and bacterial etiology of neuroinfection (Table 2).

**Figure 2:**
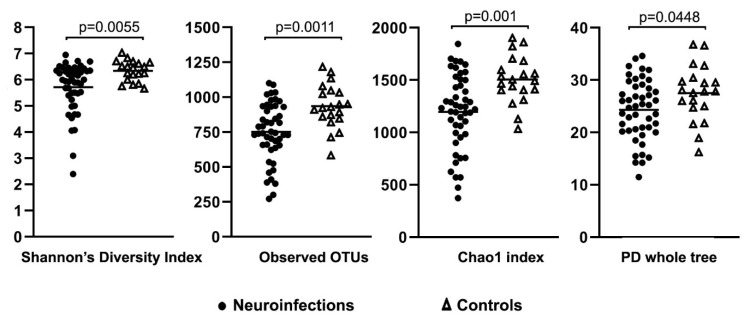
Various alpha diversity metrics in 47 patients with neuroinfection and 20 controls.

Patients with neuroinfections and controls differed with respect to some alpha diversity metrics. Thus, the Shannon’s diversity index (*p* = 0.0055) and observed OTUs (*p* = 0.0011) were significantly lower in the entire group of neuroinfections compared to controls (Figure 2). Furthermore, the Shannon’s diversity index (*p* = 0.011), observed OTUs (*p* = 0.0069), Chao1 index (*p* = 0.004), and PD whole tree index (*p* = 0.0319) were significantly lower in samples with viral etiology compared to controls.

**Figure 3:**
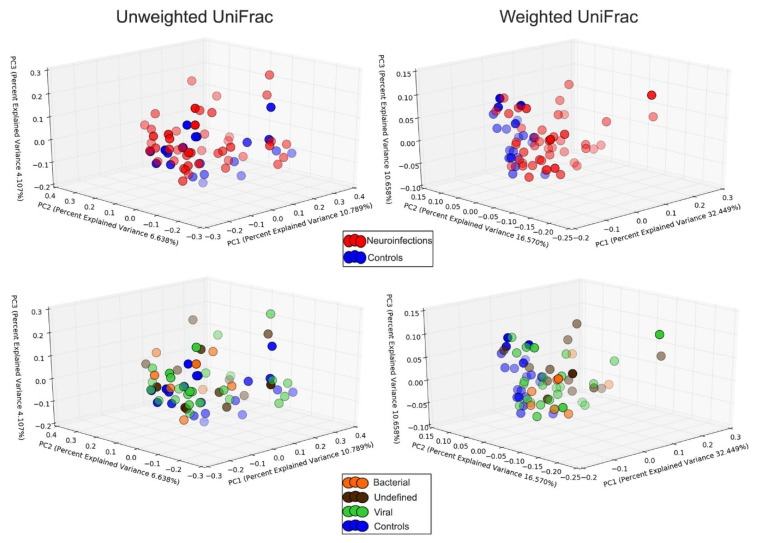
PCoA of unweighted and weighted UniFrac distances at genus level in 47 patients with neuroinfection (divided by the etiology into bacterial, viral, and undefined groups) and 20 controls.

Beta diversity, defined by principal coordinate plots (PCoA) and the analysis of similarities (ANOSIM), did not differ significantly between patients with neuroinfection and controls. Similarly, there were no differences when bacterial and viral cases were compared to each other and to controls (Figure 3).

**Figure 4:**
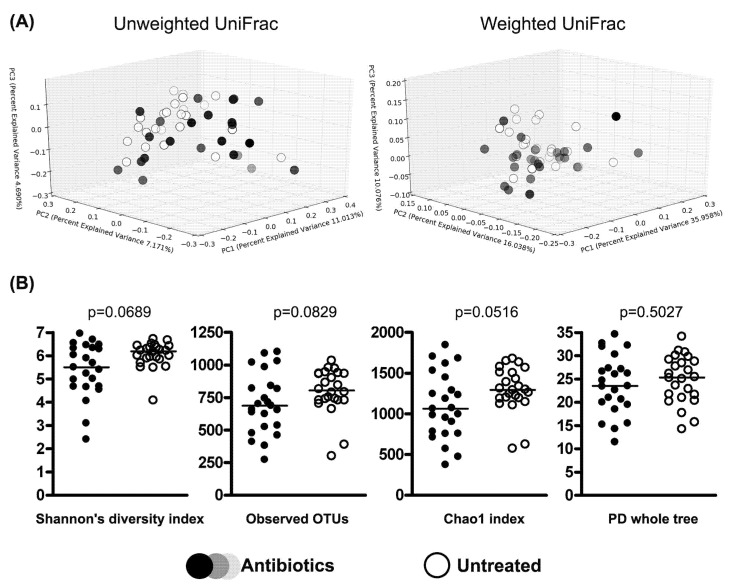
Comparison between 47 patients with neuroinfection receiving antibiotics and not receiving antibiotics. (**A**) Beta diversity—PCoA of unweighted and weighted UniFrac distances at genus level. (**B**) Alpha diversity metrics.

Comparisons of alpha and beta diversity metrics between patients receiving antibiotics and those who remained untreated did not reveal any significant differences (Figure 4). However, the Chao1 index and Shannon’s diversity index were borderline significant (*p* value was 0.0516 and 0.0689, respectively).

## 4. Discussion

Gut microbiota composition may affect a number of immune mechanisms, including lipopolysaccharide(LPS)-mediated modulation of inflammation [29], toll-like receptors (TLRs) expression [30], or changes in proinflammatory cytokines [31], which in turn could modify the course and outcome of CNS infections. Furthermore, such metabolites as occludin and claudin 5 could affect the permeability of the blood–brain barrier (BBB) and thus have an effect on the course of neuroinfection [32]. Unlike in patients suffering from chronic inflammatory maladies [33,34], changes in gut microbial composition were rarely studied in those with acute inflammatory processes. So far, alterations in the intestinal microbiome have been described in patients with acute pancreatitis [35] and acute myocardial infarction [36]. Moreover, experimental studies in animal models demonstrated a link between intestinal microbiota and the possible outcome of acute kidney injuries [37].

In our study, gut 16S rRNA profiling in patients with neuroinfection revealed significant differences in microbial composition at different taxonomic levels when compared to healthy subjects. In contrast to the negative study analyzing gut microbiota in 23 NMDAR encephalitis, we found a lower abundance of genera representing the family of *Lachnospiraceae* (*Anaerostipes, Roseburi, Lachnobacterium,* and *Lachnospira)* and genus *Clostridium* in the entire neuroinfection group, as well as in patients with viral etiology. Another 16S rRNA-based analysis of fecal microbiota in NMDAR encephalitis revealed dysbiosis in the gut microbiome represented mainly by the depletion of short-chain fatty acid (SCFA)-producing bacteria, which is similar to our study [38]. SCFAs such as butyrate, acetate, and succinate were associated with the pathophysiology of several neurological disorders, including AD, MS, PD, depression, and many others [39]. Moreover, based on a comparison between germ-free mice and those with normal gut flora, it was found that bacterial strains that produce SCFAs improve the integrity of the blood–brain barrier [40]. SCFAs also regulate microglia homeostasis, and it was reported that oral administration of acetate, propionate and butyrate is sufficient to drive microglia maturation in germ-free mice [41,42].

Alterations in specific gut microbiota were also reported in anti-leucine-rich glioma-inactivated 1 (anti-LGI1) autoimmune encephalitis patients. Similarly to our findings, genera *Roseburia* and *Lachnospira* were decreased in encephalitis patients when compared to healthy controls [43]. It should be noted that in the same study, genus *Clostridium* was more abundant in anti-LGI1 encephalitis patients than in controls [43]. However, due to the different pathogenesis of autoimmune encephalitis, the relevance of the above findings to our analysis remains unclear.

In our study, alpha diversity parameters reflecting the within-sample diversity of the gut microbiota were lower in patients with neuroinfection compared to controls. It is widely believed that increased diversity is associated with health, while its decrease is associated with dysbiosis and chronic diseases (e.g., inflammatory bowel diseases, hypertension, obesity) [33,44,45,46]. While treatment with antibiotics did not significantly affect beta diversity metrics, such alpha diversity metrics as Chao1 (*p* = 0.0516) and Shannon’s diversity index (*p* = 0.0689) were close to being statistically significant. It is likely that these differences would be more pronounced after slightly longer treatment. So far, there is little data on the effect of short-term antibiotic treatment on the human gut microbiome, as most studies concentrate on long-term sequelae and microbial recovery [47,48]. However, it was reported that 3 to 7 days of antibiotic intake might decrease the microbial load in the human gut [49]. In yet another study, Gu et al. showed that changes in alpha and beta diversity in the gut microbiota of mice exposed for four days to antibiotics occur four days after the end of treatment [50]. In another study on mice, model changes in intestinal microbiota were observed 1–5 days after the end of antibiotic treatment. These changes developed after 3 days of treatment and were highly dependable on the type of antibiotic—there were significant shifts in bacterial phyla when mice were receiving dicloxacillin and clindamycin, and only minor changes were observed when ciprofloxacin, cefotaxime, or cefuroxime were applied [51]. However, another study showed a rapid reduction in the abundance of gut bacteria in mice already on the second day of exposure to broad-spectrum antibiotics [52].

Whether alpha diversity downregulation is associated with such neurological diseases as MS and PD remains unclear, as concluded by a recent meta-analysis [53]. Similarly, studies on autoimmune encephalitis are inconclusive and contradictory. Thus, while in NMDAR encephalitis patients, alpha diversity was reported to be higher or similar to controls, in anti-LGI1 encephalitis, it was found to be lower [12,38,43]. Loss of microbial diversity can occur within just a few days due to various factors such as changes in nutrition (which are common during symptomatic diseases), hospitalization, and the administration of common drugs—and the degree of this loss may correlate with the overall disease severity [54,55,56].

## 5. Conclusions

In conclusion, in our analysis of the gut microbiome, some particular bacterial taxa (*Clostridium, Anaerostipes, Lachnobacterium, Lachnospira,* and *Roseburia*) were decreased in patients with neuroinfection compared to controls. Similarly, alpha diversity metrics showed lower within-sample diversity in these patients. The cause of these changes is unclear and could be due to the inflammatory process accompanying the disease; however, the effect of diet modification and/or hospitalization itself cannot be excluded. Short-term (1–3 day) antibiotic treatment did not affect beta diversity, but alpha diversity metrics were close to being statistically significant.

## Figures and Tables

**Table 1:** Demographical, clinical, and laboratory data in 47 patients with neuroinfection and 20 controls.

Neuroinfections	Number (%)			
	All Patients	Viral	Bacterial	Undefined
Nº of patients	47	26 (55.32)	8 (17.02)	13 (27.66)
Male	23 (48.94)	13 (50)	4 (50)	6 (46.15)
Female	24 (51.06)	13 (50)	4 (50)	7 (53.85)
Age, Mean	43	41	50	44
**Symptoms and signs**	**Number (%)**			
	**All patients**	**Viral**	**Bacterial**	**Undefined**
Fever	31 (65.96)	19 (73.08)	6 (75)	6 (46.15)
Headache	37 (78.72)	22 (84.62)	6 (75)	9 (69.26)
Altered mental status	11 (23.40)	2 (7.69)	4 (50)	5 (38.46)
Loss of consciousness	6 (12.76)	2 (7.69)	1 (50)	3 (23.08)
Seizures of epilepsy	4 (8.51)	1 (3.85)	0	3 (23.08)
Focal neurologic signs	19 (40.43)	13 (50)	6 (75)	0
**CSF analysis (ref. values)**	**Mean value/standard deviation**			
	**All patients**	**Viral**	**Bacterial**	**Undefined**
Total cell count, cells/µL (≤5)	190/238	155/120	619/379	64/114
% of lymphocytes	63/30.46	69/27	20/6	50/37
Chlorides, mmol/L (>117)	121.72/4.15	121.61/3.22	119.00/5.61	123.49/3.91
Protein, g/L (0.15–0.45)	1.51/1.62	1.09/0.89	2.51/1.87	1.77/2.23
L-Lactic acid, mmol/L (≤2.1)	2.23/0.99	2.15/0.61	4.94/1.33	1.93/0.77
Glucose, mmol/L (2.4–4.7)	2.80/0.87	2.97/0.35	1.64/0.73	3.28/1.02
**Blood test (ref. values)**	**Mean value/standard deviation**			
	**All patients**	**Viral**	**Bacterial**	**Undefined**
WBC, ×10^3^/µL (–10)	10.65/4.04	9.05/2.72	14.74/3.42	10.87/4.48
RBC, ×10^6^/µL (4.5–5.9)	4.56/0.57	4.65/0.51	3.99/0.75	4.63/0.44
Platelet count, ×10^3^/µL (150–450)	238.48/63.80	233.87/52.42	204.50/58.14	265.80/70.69
CRP, mg/L (<5)	40.84/63.04	13.33/8.55	120.37/86.66	19.77/18.77
**Treatment**	**Number (%)**			
	**All patients**	**Viral**	**Bacterial**	**Undefined**
Antibiotics	23 (48.94)	9 (34.62)	8 (100)	6 (46.15)
Antiviral drugs (Aciclovir)	15 (31.9)	10 (38.46)	0	5 (38.46)
Antiepileptic Drugs	7 (14.89)	2 (7.69)	1 (12.50)	4 (30.77)
**Control group (%)**				
Nº of controls	20			
Male	10 (50)			
Female	10 (50)			
Age, Mean	43			

**Table 2:** Differences at taxonomic ranks between patients with neuroinfections and healthy controls.

Taxonomy		Controls (*n* = 20) vs.		
		Neuroinfections (*n* = 47)	Bacterial (*n* = 8)	Viral (*n* = 26)
Phylum	*p__ Bacillota*	0.015	0.037	0.037
	*p__ Actinomycetota*		0.037	
	*p__ Verrucomicrobiota*			0.030
Class	*p__ Bacillota;c__Clostridia*	0.026	0.013	0.026
	*p__ Actinomycetota;c__Coriobacteriia*		0.013	
	*p__ Verrucomicrobiota;c__Verrucomicrobiae*			0.039
Order	*p__ Bacillota;c__Clostridia;o__Clostridiales*	0.043	0.021	
	*p__ Actinomycetota;c__Coriobacteriia; o__Coriobacteriales*		0.021	
Family	*p__ Bacillota;c__Clostridia;o__Clostridiales;f__Eubacteriaceae*			0.045
	*p__ Verrucomicrobiota;c__Verrucomicrobiae; o__Verrucomicrobiales;f__Verrucomicrobiaceae*			0.045
Genus	*p__ Bacillota;c__Clostridia;o__Clostridiales;f__Clostridiaceae;g__Clostridium*	0.025		0.040
	*p__ Bacillota;c__Clostridia;o__Clostridiales;f__Lachnospiraceae;g__Anaerostipes*	0.025		0.040
	*p__ Bacillota;c__Clostridia;o__Clostridiales;f__Lachnospiraceae;g__Lachnobacterium*	0.025		0.040
	*p__ Bacillota;c__Clostridia;o__Clostridiales;f__Lachnospiraceae;g__Lachnospira*	0.025		0.040
	*p__ Bacillota;c__Clostridia;o__Clostridiales;f__Lachnospiraceae;g__Roseburia*	0.025		0.040

## Data Availability

The datasets generated during the current study are available in the Sequence Read Archive (SRA accession: BioProject PRJNA836385).

## References

[1] Scheperjans F., Aho V., Pereira P.A., Koskinen K., Paulin L., Pekkonen E., Haapaniemi E., Kaakkola S., Eerola-Rautio J., Pohja M. (2015). Gut microbiota are related to Parkinson’s disease and clinical phenotype. Mov. Disord..

[2] Shen T., Yue Y., He T., Huang C., Qu B., Lv W., Lai H.Y. (2021). The Association Between the Gut Microbiota and Parkinson’s Disease, a Meta-Analysis. Front. Aging Neurosci..

[3] van Olst L., Roks S.J.M., Kamermans A., Verhaar B.J.H., van der Geest A.M., Muller M., van der Flier W.M., de Vries H.E. (2021). Contribution of Gut Microbiota to Immunological Changes in Alzheimer’s Disease. Front. Immunol..

[4] Miyake S., Kim S., Suda W., Oshima K., Nakamura M., Matsuoka T., Chihara N., Tomita A., Sato W., Kim S.W. (2015). Dysbiosis in the Gut Microbiota of Patients with Multiple Sclerosis, with a Striking Depletion of Species Belonging to Clostridia XIVa and IV Clusters. PLoS ONE.

[5] Cantoni C., Lin Q., Dorsett Y., Ghezzi L., Liu Z., Pan Y., Chen K., Han Y., Li Z., Xiao H. (2022). Alterations of host-gut microbiome interactions in multiple sclerosis. EBioMedicine.

[6] McGuinness A.J., Davis J.A., Dawson S.L., Loughman A., Collier F., O’Hely M., Simpson C.A., Green J., Marx W., Hair C. (2022). A systematic review of gut microbiota composition in observational studies of major depressive disorder, bipolar disorder and schizophrenia. Mol. Psychiatry.

[7] Garcia-Gutierrez E., Narbad A., Rodriguez J.M. (2020). Autism Spectrum Disorder Associated With Gut Microbiota at Immune, Metabolomic, and Neuroactive Level. Front. Neurosci..

[8] Li Y., Zhang B., Zhou Y., Wang D., Liu X., Li L., Wang T., Zhang Y., Jiang M., Tang H. (2020). Gut Microbiota Changes and Their Relationship with Inflammation in Patients with Acute and Chronic Insomnia. Nat. Sci. Sleep.

[9] Voigt R.M., Forsyth C.B., Green S.J., Engen P.A., Keshavarzian A. (2016). Circadian Rhythm and the Gut Microbiome. Int. Rev. Neurobiol..

[10] Wang Y., Wang Z., Wang Y., Li F., Jia J., Song X., Qin S., Wang R., Jin F., Kitazato K. (2018). The Gut-Microglia Connection: Implications for Central Nervous System Diseases. Front. Immunol..

[11] Gubert C., Gasparotto J., Morais L.M. (2022). Convergent pathways of the gut microbiota-brain axis and neurodegenerative disorders. Gastroenterol. Rep..

[12] Herken J., Bang C., Ruhlemann M.C., Finke C., Klag J., Franke A., Pruss H. (2019). Normal gut microbiome in NMDA receptor encephalitis. Neurol. Neuroimmunol. Neuroinflamm..

[13] Kennedy P.G.E., Quan P.L., Lipkin W.I. (2017). Viral Encephalitis of Unknown Cause: Current Perspective and Recent Advances. Viruses.

[14] Potharaju N.R. (2012). Incidence Rate of Acute Encephalitis Syndrome without Specific Treatment in India and Nepal. Indian J. Community Med..

[15] Glaser C.A., Gilliam S., Schnurr D., Forghani B., Honarmand S., Khetsuriani N., Fischer M., Cossen C.K., Anderson L.J. (2003). In search of encephalitis etiologies: Diagnostic challenges in the California Encephalitis Project, 1998–2000. Clin. Infect. Dis..

[16] Perlejewski K., Bukowska-Osko I., Rydzanicz M., Pawelczyk A., Caraballo Corts K., Osuch S., Paciorek M., Dzieciatkowski T., Radkowski M., Laskus T. (2020). Next-generation sequencing in the diagnosis of viral encephalitis: Sensitivity and clinical limitations. Sci. Rep..

[17] Glaser C.A., Honarmand S., Anderson L.J., Schnurr D.P., Forghani B., Cossen C.K., Schuster F.L., Christie L.J., Tureen J.H. (2006). Beyond viruses: Clinical profiles and etiologies associated with encephalitis. Clin. Infect. Dis..

[18] Granerod J., Ambrose H.E., Davies N.W.S., Clewley J.P., Walsh A.L., Morgan D., Cunningham R., Zuckerman M., Mutton K.J., Solomon T. (2010). Causes of encephalitis and differences in their clinical presentations in England: A multicentre, population-based prospective study. Lancet Infect. Dis..

[19] Klindworth A., Pruesse E., Schweer T., Peplies J., Quast C., Horn M., Glockner F.O. (2013). Evaluation of general 16S ribosomal RNA gene PCR primers for classical and next-generation sequencing-based diversity studies. Nucleic Acids Res..

[20] Andrews S. (2010). FastQC: A Quality Control Tool for High Throughput Sequence Data. http://www.bioinformatics.babraham.ac.uk/projects/fastqc.

[21] Bolger A.M., Lohse M., Usadel B. (2014). Trimmomatic: A flexible trimmer for Illumina sequence data. Bioinformatics.

[22] Bushnell B., Rood J., Singer E. (2017). BBMerge—Accurate paired shotgun read merging via overlap. PLoS ONE.

[23] Caporaso J.G., Kuczynski J., Stombaugh J., Bittinger K., Bushman F.D., Costello E.K., Fierer N., Pena A.G., Goodrich J.K., Gordon J.I. (2010). QIIME allows analysis of high-throughput community sequencing data. Nat. Methods.

[24] Aronesty E. (2011). Ea-Utils: “Command-Line Tools for Processing Biological Sequencing Data”. https://github.com/ExpressionAnalysis/ea-utils.

[25] Haas B.J., Gevers D., Earl A.M., Feldgarden M., Ward D.V., Giannoukos G., Ciulla D., Tabbaa D., Highlander S.K., Sodergren E. (2011). Chimeric 16S rRNA sequence formation and detection in Sanger and 454-pyrosequenced PCR amplicons. Genome Res..

[26] Paulson J.N., Stine O.C., Bravo H.C., Pop M. (2013). Differential abundance analysis for microbial marker-gene surveys. Nat. Methods.

[27] Dabdoub S.M., Fellows M.L., Paropkari A.D., Mason M.R., Huja S.S., Tsigarida A.A., Kumar P.S. (2016). PhyloToAST: Bioinformatics tools for species-level analysis and visualization of complex microbial datasets. Sci. Rep..

[28] McMurdie P.J., Holmes S. (2013). phyloseq: An R package for reproducible interactive analysis and graphics of microbiome census data. PLoS ONE.

[29] Noori M.S., Courreges M.C., Bergmeier S.C., McCall K.D., Goetz D.J. (2020). Modulation of LPS-induced inflammatory cytokine production by a novel glycogen synthase kinase-3 inhibitor. Eur. J. Pharmacol..

[30] Takeda K., Akira S. (2001). Roles of Toll-like receptors in innate immune responses. Genes Cells.

[31] Bailey M.T., Dowd S.E., Galley J.D., Hufnagle A.R., Allen R.G., Lyte M. (2011). Exposure to a social stressor alters the structure of the intestinal microbiota: Implications for stressor-induced immunomodulation. Brain Behav. Immun..

[32] Jia W., Lu R., Martin T.A., Jiang W.G. (2014). The role of claudin-5 in blood-brain barrier (BBB) and brain metastases (review). Mol. Med. Rep..

[33] Gong D., Gong X., Wang L., Yu X., Dong Q. (2016). Involvement of Reduced Microbial Diversity in Inflammatory Bowel Disease. Gastroenterol Res. Pract..

[34] Wehedy E., Shatat I.F., Al Khodor S. (2021). The Human Microbiome in Chronic Kidney Disease: A Double-Edged Sword. Front. Med..

[35] Tan C., Ling Z., Huang Y., Cao Y., Liu Q., Cai T., Yuan H., Liu C., Li Y., Xu K. (2015). Dysbiosis of Intestinal Microbiota Associated With Inflammation Involved in the Progression of Acute Pancreatitis. Pancreas.

[36] Han Y., Gong Z., Sun G., Xu J., Qi C., Sun W., Jiang H., Cao P., Ju H. (2021). Dysbiosis of Gut Microbiota in Patients with Acute Myocardial Infarction. Front. Microbiol..

[37] Gong J., Noel S., Pluznick J.L., Hamad A.R.A., Rabb H. (2019). Gut Microbiota-Kidney Cross-Talk in Acute Kidney Injury. Semin. Nephrol..

[38] Gong X., Liu X., Li C., Chen C., Lin J., Li A., An D., Zhou D., Hong Z. (2019). Alterations in the human gut microbiome in anti-N-methyl-D-aspartate receptor encephalitis. Ann. Clin. Transl. Neurol..

[39] Mirzaei R., Bouzari B., Hosseini-Fard S.R., Mazaheri M., Ahmadyousefi Y., Abdi M., Jalalifar S., Karimitabar Z., Teimoori A., Keyvani H. (2021). Role of microbiota-derived short-chain fatty acids in nervous system disorders. Biomed. Pharmacother..

[40] Braniste V., Al-Asmakh M., Kowal C., Anuar F., Abbaspour A., Toth M., Korecka A., Bakocevic N., Ng L.G., Kundu P. (2014). The gut microbiota influences blood-brain barrier permeability in mice. Sci. Transl. Med..

[41] Erny D., Hrabe de Angelis A.L., Jaitin D., Wieghofer P., Staszewski O., David E., Keren-Shaul H., Mahlakoiv T., Jakobshagen K., Buch T. (2015). Host microbiota constantly control maturation and function of microglia in the CNS. Nat. Neurosci..

[42] Silva Y.P., Bernardi A., Frozza R.L. (2020). The Role of Short-Chain Fatty Acids From Gut Microbiota in Gut-Brain Communication. Front. Endocrinol..

[43] Ma X., Ma L., Wang Z., Liu Y., Long L., Ma X., Chen H., Chen Z., Lin X., Si L. (2020). Clinical Features and Gut Microbial Alterations in Anti-leucine-rich Glioma-Inactivated 1 Encephalitis-A Pilot Study. Front. Neurol..

[44] Pickard J.M., Zeng M.Y., Caruso R., Nunez G. (2017). Gut microbiota: Role in pathogen colonization, immune responses, and inflammatory disease. Immunol. Rev..

[45] Jin L., Shi X., Yang J., Zhao Y., Xue L., Xu L., Cai J. (2021). Gut microbes in cardiovascular diseases and their potential therapeutic applications. Protein Cell.

[46] Davis C.D. (2016). The Gut Microbiome and Its Role in Obesity. Nutr. Today.

[47] Palleja A., Mikkelsen K.H., Forslund S.K., Kashani A., Allin K.H., Nielsen T., Hansen T.H., Liang S., Feng Q., Zhang C. (2018). Recovery of gut microbiota of healthy adults following antibiotic exposure. Nat. Microbiol..

[48] Kwon Y., Cho Y.-S., Lee Y.-M., Kim S.-j., Bae J., Jeong S.-J. (2022). Changes to Gut Microbiota Following Systemic Antibiotic Administration in Infants. Antibiotics.

[49] Panda S., El khader I., Casellas F., Lopez Vivancos J., Garcia Cors M., Santiago A., Cuenca S., Guarner F., Manichanh C. (2014). Short-term effect of antibiotics on human gut microbiota. PLoS ONE.

[50] Gu S.L., Gong Y.W., Zhang J.Y., Chen Y.B., Wu Z.J., Xu Q.M., Fang Y.H., Wang J.X., Tang L.L. (2020). Effect of the Short-Term Use of Fluoroquinolone and beta-Lactam Antibiotics on Mouse Gut Microbiota. Infect. Drug Resist..

[51] Hertz F.B., Budding A.E., van der Lugt-Degen M., Savelkoul P.H., Lobner-Olesen A., Frimodt-Moller N. (2020). Effects of Antibiotics on the Intestinal Microbiota of Mice. Antibiotics.

[52] Yao J., Carter R.A., Vuagniaux G., Barbier M., Rosch J.W., Rock C.O. (2016). A Pathogen-Selective Antibiotic Minimizes Disturbance to the Microbiome. Antimicrob. Agents Chemother..

[53] Plassais J., Gbikpi-Benissan G., Figarol M., Scheperjans F., Gorochov G., Derkinderen P., Cervino A.C.L. (2021). Gut microbiome alpha-diversity is not a marker of Parkinson’s disease and multiple sclerosis. Brain Commun..

[54] McDonald D., Ackermann G., Khailova L., Baird C., Heyland D., Kozar R., Lemieux M., Derenski K., King J., Vis-Kampen C. (2016). Extreme Dysbiosis of the Microbiome in Critical Illness. mSphere.

[55] Ravi A., Halstead F.D., Bamford A., Casey A., Thomson N.M., van Schaik W., Snelson C., Goulden R., Foster-Nyarko E., Savva G.M. (2019). Loss of microbial diversity and pathogen domination of the gut microbiota in critically ill patients. Microb. Genom..

[56] Zaborin A., Smith D., Garfield K., Quensen J., Shakhsheer B., Kade M., Tirrell M., Tiedje J., Gilbert J.A., Zaborina O. (2014). Membership and behavior of ultra-low-diversity pathogen communities present in the gut of humans during prolonged critical illness. mBio.

